# Awareness and resilience among Indian adolescents on cyberbullying

**DOI:** 10.6026/973206300210334

**Published:** 2025-03-31

**Authors:** Albeenasiril Marianathan, Theranirajan Ethiraj, Shankar Shanmugam Rajendran, Venkatesh Madhan Kumar, Anbalagan Marudan, Jayalakshmi Lakshmanan, Geetha Rajamoorthy

**Affiliations:** 1 Department of Psychiatric Nursing, College of Nursing, Madras Medical College, Chennai, The TN Dr MGR Medical University, Chennai, Tamil Nadu, India; 2 Department of Pediatrics, Madras Medical College, The TN Dr MGR Medical University, Chennai, Tamil Nadu, India; 3 Department of Pediatric Nursing, College of Nursing, Madras Medical College, The TN Dr MGR Medical University, Chennai, Tamil Nadu, India; 4 Department of Psychiatry, Institute of Mental Health, Madras Medical College, The TN Dr MGR Medical University, Chennai, Tamil Nadu, India; 5 Department of Child Health Nursing, Madras Medical College, the TN Dr MGR Medical University, Chennai, Tamil Nadu, India

**Keywords:** Cyberbullying, adolescents, cognizance sessions, coping strategies, mental health

## Abstract

Cyberbullying is prevalent among adolescents. This often occurs through online harassment on social media. Therefore, it is of interest to assess the impact of cognizance sessions on adolescents' awareness and its coping strategies. Hence, 160 adolescents were divided into experimental and control groups using a quasi-experimental design. Results indicated significant improvements in the experimental group's awareness and coping scores after educational sessions. These findings demonstrate the effectiveness of such interventions, emphasizing the need for further research with larger samples.

## Background:

The rise of social media platforms has dramatically altered adolescent behavior, often contributing to an increase in cyberbullying incidents. Defined as online harassment, cyberbullying includes actions such as the dissemination of embarrassing images, derogatory comments and rumor-spreading [1]. The psychological consequences for victims can be profound, adversely affecting their mental health, relationships and overall quality of life. Recognizing the prevalence and effects of cyberbullying, particularly among teenagers, is critical for developing effective intervention strategies [2]. Cyberbullying is characterized by the systematic abuse of power through technological means. Victims may include those who are directly targeted, participants in the harassment, or bystanders who witness the abuse. The complexity of these dynamics necessitates a comprehensive understanding of the phenomenon [3]. Worldwide studies suggest that cyberbullying affects as many as 70% of adolescents. A 2022 survey revealed that over half of American youth reported experiencing some form of cyberbullying. Additionally, a troubling aspect involves racially motivated bullying, with 75% of these incidents occurring on platforms like Facebook, highlighting the need for targeted interventions [4]. Research conducted within the country indicates a significant rise in cyberbullying incidents among both male and female adolescents. This alarming trend underscores the need for effective educational programs and support systems to address the issue in schools and communities [5]. A local study in Chennai revealed that 63% of adolescents aged 16-18 identified as victims of cyberbullying, highlighting a vulnerable demographic in Tamil Nadu. This significant prevalence calls for urgent attention to address the mental health and social implications for these young individuals [6]. Cyberbullying, characterized by an imbalance of power and repetitive hostile behavior, poses a significant public health concern for adolescents, particularly in the context of increased Internet and electronic device usage [7]. Victims often experience heightened anxiety, depression, loneliness and a range of other physical and psychological issues, which can adversely affect their social and academic performance. Research indicates a wide prevalence of cyberbullying among teens, with rates of victimization ranging from 1% to 72%. Numerous studies have highlighted its detrimental impact on mental health and coping strategies [8]. Effective prevention requires increased awareness and knowledge of cyberbullying among both adolescents and parents, with health professionals, including nurses, playing a crucial role in educational efforts. Various intervention programs have demonstrated success in enhancing awareness and coping skills, underscoring the importance of preventative measures that cultivate empathy, promote anti-bullying attitudes and equip victims with self-defense tactics [9]. Therefore, it is of interest to describe the awareness and coping mechanisms related to cyberbullying among Indian adolescents at Madras Medical College's School of Nursing and College of Nursing in Chennai.

## Methodology:

## Statement of the problem:

The effect of cyberbullying cognisance sessions on realisation and coping strategies among adolescents in the selected colleges in Chennai is of interest.

## Operational definitions:

## Effect:

Improvement in awareness and coping strategies related to cyberbullying.

## Cyberbullying:

Intentional harassment carried out through digital means.

## Cognizance session:

Educational sessions focused on increasing knowledge about cyberbullying.

## Realization:

The level of understand and awareness of cyberbullying.

## Coping strategies:

Methods and techniques employed to manage stress and emotional fallout related to cyberbullying.

## Adolescents:

Individuals aged 17-19 years participating in the study.

## Assumptions:

Adolescents may exhibit low levels of realization and coping strategies regarding cyberbullying. Cognizance sessions may enhance awareness and coping mechanisms among nursing students.

## Hypotheses:

H1: Significant differences exist between pre-test and post-test scores in both the experimental and control groups.

H2: A significant association exists between the experimental group's post-test scores and demographic variables.

## Delimitations:

The study explicitly targets adolescents at the MMC School and College of Nursing. Data collection will take place over four weeks and involve a limited sample size.

## Conceptual framework:

The framework for this study is constructed based on Wiedenbach's Helping Art of Clinical Nursing Theory:

## Central purpose:

To assess the impact of cognizance sessions on awareness and coping strategies related to cyberbullying.

## Prescription:

Conduct educational sessions focusing on the various aspects of cyberbullying.

## Participants:

The researcher (agent) and adolescents (recipients) will be used to improve realization and coping scores.

## Research approach:

A quantitative research approach was employed to systematically evaluate the effects of cyberbullying cognizance sessions on adolescents' realization and coping strategies.

## Research design:

## A quasi-experimental design was implemented, structured as follows:

## Experimental group:

Pre-test (O1) → Intervention (X) → Post-test (O2)

## Control group:

Pre-test (O1) → No intervention → Post-test (O2)

## Variables:

## Independent variable:

## Cyberbullying cognizance sessions:

## Dependent variables:

Levels of realization and coping strategies

## Extraneous variables:

Age, sex, religion, parental education, location and other demographic factors.

## Research setting:

The study was conducted at Madras Medical College School of Nursing and College of Nursing in Chennai, India.

## Study population:

The target population comprised adolescents enrolled at the School and College of Nursing.

## Sample size:

160 adolescents participated in the study, with 80 assigned to each group based on prior sample size calculations.

## Sampling technique:

A non-randomized convenience sampling technique was utilized to select participants.

## Sampling criteria:

## Inclusion criteria:

Adolescents aged 17 to 19 who consented to participate.

## Exclusion criteria:

Adolescents are unavailable for participation or are enrolled in other educational programs.

## Data collection instruments:

Data were collected using two sections:

## Demographic variables:

Information regards participants' demographics.

## Cyberbullying realization and coping scales:

Standardized instruments measure adolescents' awareness of cyberbullying and their coping strategies.

## Reliability and validity:

The reliability of the scales was established with a Cronbach's alpha of 0.72, indicating acceptable internal consistency. Content validity was ensured through peer reviews.

## Ethical considerations:

Ethical approval was obtained from the Madras Medical College Institutional Ethics Committee. Researchers ensured compliance with ethical guidelines throughout the study, including informed consent from all participants.

## Results:

The study revealed that 41.25% of adolescents in the experimental group and 40.00% in the control group were 18. The sample predominantly consisted of female participants (90.00% experimental and 87.50% control) and Hindus (76.25% experimental and 73.75% control) (Figure 1 - see PDF). Pre-test scores indicated that 26.25% of the experimental group had low realization scores, while 23.75% in the control group exhibited similar results. Post-test evaluations demonstrated that 67.50% of the experimental group achieved high realization scores compared to only 16.25% in the control group. Regarding coping strategies, 72.50% of the experimental group demonstrated good scores post-intervention, contrasting sharply with just 12.50% in the control group (Figure 2 - see PDF). Statistical analysis confirmed significant differences between the groups (p < 0.05), affirming that the intervention effectively improved both realization and coping strategies related to cyberbullying (Table 1) (Table 2).

## Discussion:

This study highlights the crucial impact of cognizance sessions on enhancing adolescents' awareness of cyberbullying and their coping abilities. The study demonstrated a significant improvement in realization and coping scores among adolescents in the experimental group compared to the control group. Post-test results showed that 67.50% of participants in the experimental group achieved a high level of realization. At the same time, none had poor coping scores, contrasting with the control group, where 83.75% reported moderate realization and 5.00% had poor coping scores. These findings align with Yurdakul *et al.* [10], which also indicated significant enhancements in awareness and coping strategies post-intervention, supporting the efficacy of cyberbullying awareness programs in promoting adolescents' skills. The study demonstrated significant improvements in realization and coping scores for the experimental group compared to the control group. The experimental group showed a 22.21% increase in realization scores and a 25.63% increase in coping scores, while the control group had only 0.87% and 2.03% gains, respectively. These results indicate the effectiveness of awareness sessions, leading to the acceptance of the alternative hypothesis (H1).

The study found a significant association between demographic variables and post-test scores in the experimental group, where higher education and urban residency correlated with elevated realization and coping scores. In contrast, the control group exhibited no high scores. These findings align with Qiu *et al.* [11] and Arseneault *et al.* [12], identifying demographic predictors of bullying and victimization. The results confirmed the effectiveness of the intervention, leading to the acceptance of the alternative hypothesis (H2). The findings of this study have several important implications for nursing education, practice, management and research. In nursing education, it is essential to incorporate topics related to cyberbullying into the curricula, along with workshops aimed at helping students recognize signs of distress among affected adolescents. In nursing practice, educational sessions should be conducted to address cyberbullying, equipping healthcare professionals with strategies to provide emotional support to those impacted. Additionally, nursing management must focus on developing and enforcing comprehensive policies regarding cyberbullying and providing training for staff to address these issues effectively. Lastly, nursing research should be encouraged to explore further studies that investigate effective interventions and their implications across diverse populations, ultimately contributing to a more informed and supportive healthcare environment for adolescents facing cyberbullying. This study demonstrates the significant positive impact of cognizance sessions on enhancing adolescents' awareness and coping strategies regarding cyberbullying. Our findings are consistent with those of Arseneault [13] and Zhu *et al.* [14], who found that interventions aimed at improving awareness, can significantly mitigate the negative effects of cyberbullying on adolescents. Similar to the results presented by Arseneault, where interventions targeted bullying awareness, our study showed a considerable increase in realization and coping scores among the experimental group, indicating that awareness and coping strategies can be improved with structured educational sessions. In this study, a significant increase in realization and coping abilities was observed in the experimental group, with a 22.21% improvement in realization scores and a 25.63% improvement in coping scores. These results align with the study by Zhu *et al.* (2021), where awareness programs focused on bullying showed a notable effect in enhancing the coping mechanisms of adolescents, particularly in recognizing and managing cyberbullying. It is crucial to note that both studies emphasize the role of targeted interventions, which support the idea that structured sessions provide adolescents with the tools to combat the detrimental effects of online harassment effectively. Furthermore, the significant improvements observed in the experimental group as compared to the control group highlight the need for incorporating such awareness programs into educational curricula. This is in line with the findings of Vijayarani *et al.* (2024), [15], who suggested that educational institutions should integrate programs that promote awareness and resilience against cyberbullying. The significant demographic differences observed in the experimental group, such as higher realization scores among students with higher parental education, suggest that demographic variables play a role in how effectively adolescents respond to such interventions. This finding echoes the research conducted by Qiu *et al.* [12] which identified similar trends in the relationship between demographic factors and susceptibility to cyberbullying. Overall, the study underscores the effectiveness of awareness programs in improving adolescents' coping strategies and awareness of cyberbullying, supporting the hypothesis that such interventions are a critical component in tackling cyberbullying. The significant improvements seen in the experimental group provide strong evidence for the continued development and implementation of educational programs, particularly in nursing schools, to prepare adolescents to manage the challenges posed by cyberbullying. These findings contribute to the growing body of research on cyberbullying interventions, reinforcing the importance of raising awareness and fostering resilience among adolescents. Further research involving a larger and more diverse sample would help in refining strategies for mitigating the impact of cyberbullying in various cultural contexts.

## Limitations:

The study acknowledges limitations, including a small sample size that may restrict the generalizability of findings and potential selection bias due to the convenience sampling method.

## Conclusion:

Cyberbullying poses a significant risk to the psychological well-being of adolescents. Hence, the critical role of collaborative efforts among educational institutions, healthcare professionals and parents in developing targeted intervention programs is highlighted. Thus, a multifaceted approach is essential to effectively combat the adverse effects of cyberbullying, highlighting the need for on-going research to inform more effective strategies.

## Figures and Tables

**Table 1:** Comparison of realization score

**Realization score**	**Group**		**Mean difference**	**Student independent t-test**	
	**Experimental**	**Control**			
	**Mean**	**SD**	**Mean**	**SD**	
Pre-test	28.36	4.82	28.98	4.66	t = 0.82 p = 0.42 (NS)
Post-test	41.69	5.92	29.5	4.65	t = 14.48 p = 0.001*** (S)

**Table 2:** Comparison of coping score

**Test**	**Experimental Mean (SD)**	**Control Mean (SD)**	**t-value**	**p-value**
Pre-test	18.29 (3.55)	18.59 (5.00)	0.44	0.66 (NS)
Post-test	27.26 (3.90)	19.30 (4.33)	12.22	0.001*** (S)
